# Three new species of *Stenomesius* Westwood (Hymenoptera, Eulophidae) from China, with a key to Chinese species

**DOI:** 10.3897/zookeys.1062.67487

**Published:** 2021-10-12

**Authors:** Jun-Jie Fan, Cheng-De Li

**Affiliations:** 1 School of Forestry, Northeast Forestry University, Harbin, 150040, China Northeast Forestry University Harbin China

**Keywords:** Chalcidoidea, Eulophinae, parasitoid, taxonomy

## Abstract

Three new species of *Stenomesius* Westwood, *S.guanshanensis***sp. nov.**, *S.hani***sp. nov.**, and *S.harbinensis***sp. nov.**, are described from China. A key to all species of the genus *Stenomesius* in China is provided.

## Introduction

The genus *Stenomesius* (Hymenoptera, Eulophidae) was erected by Westwood (1833) based on European species *S.pulchellus* Westwood and *S.maculatus* Westwood. *Stenomesiuspulchellus* was subsequently designated as the type species by Westwood (1840), but *S.pulchellus* and *S.maculatus* were both synonymized with *S.rufescens* (Retzius). Noyes (2021) listed 21 species in this genus, but *S.aspidicola* Ashmead is a misspelling of *S.aphidicola* Ashmead and *S.pantnagarensis* Agnihotri and Khan was synonymized with *S.orientalis* Agnihotri and Khan (Agnihotri and Khan 2004; Khan et al. 2005), so currently the genus contains 19 valid species. Only two species were known from China: *S.japonicus* (Ashmead) and *S.maculatus* Liao (Bouček 1977; Liao et al. 1987; Zhu and Huang 2001, 2002). Most species of the genus are parasitoids of Lepidoptera, and recorded host families include Gelechiidae, Lyonettiidae, Glyphipterygidae, Tortricidae, Pyralidae, and Noctuidae (Bouček 1988). One specimen from Sulawesi (Indonesia 1949) is labelled as having been reared from eggs of *Scirphophagainnotata* (Walker), the white rice borer, but this record needs confirmation (Bouček 1988).

This study describes three new species of the genus *Stenomesius* and provides a key to all species of the genus distributed in China.

## Material and methods

All specimens were collected by sweeping or yellow pan trapping, and they were dissected and mounted in Canada balsam on slides following the method of Noyes (1982), or mounted on a card. Photos were taken with a digital CCD camera attached to an Olympus BX51 compound microscope or Aosvi AO-HK830-5870T digital microscope. Measurements were made using an eyepiece reticle, or using the ruler tool in Adobe Photoshop 2020.

Terminology follows the Hymenoptera Anatomy Consortium (2021), and the following abbreviations are used: **F1–4** = flagellomeres 1–4; **MV** = marginal vein; **OOL** = minimum distance between lateral ocellus and eye margin; **PMV** = postmarginal vein; **POL** = minimum distance between lateral ocelli; **SMV** = submarginal vein; **STV** = stigmal vein; **T1–7** = gastral tergites 1–7. All type material is deposited in the insect collections at Northeast Forestry University (**NEFU**), Harbin, China.

## Taxonomy

### Stenomesius

Taxon classificationAnimaliaHymenopteraEulophidae

Westwood, 1833

B298E3A4-781D-5EA9-A7E7-6974AAC4A6F8

Stenomesius Westwood, 1833: 343. Type species: Stenomesiuspulchellus Westwood, by subsequent designation of Westwood 1839: 73.
Euryscotolinx Girault, 1913: 266. Type species: Euryscotolinxguttativertex Girault, by original designation and monotypy. [Synonymised with Stenomesius Westwood by Bouček 1977: 401].
Stenelachistus Masi, 1917: 201. Type species: Stenelachistusimpressus Masi, by subsequent designation of Gahan and Fagan 1923: 136. [Synonymised with Stenomesius Westwood by Bouček, 1977: 401].
Nioro Risbec, 1951: 25. Type species: Nioroelegantula Risbec, by monotypy. [Synonymised with Stenomesius Westwood by Bouček 1977: 401].

#### Diagnosis.

The genus can be easily distinguished from other eulophine genera by the following combination of characters: female funicle 4-segmented and club 2–3-segmented; mandible developed; pronotum without transverse carina; scutellum with sublateral grooves; propodeum medially with a strong X- or H-shaped carinae; hind tibial spurs normal; petiole shorter than hind coxa, gaster usually elongate.

The genus *Stenomesius* is close to the genus *Stenopetius* Bouček and *Euplectromorpha* Girault in having similar type of propodeum with H- or X-shaped carinae, but it differs from *Stenopetius* Bouček in having: 1) female funicle 4-segmented (5-segmented in *Stenopetius* Bouček); 2) pronotum without transverse carina (with distinct transverse carina in *Stenopetius* Bouček); 3) petiole shorter than hind coxa (longer than hind coxa in *Stenopetius* Bouček). It differs from *Euplectromorpha* in having hind tibia normal, at least with one spur distinctly longer than first hind tarsal segment in *Euplectromorpha*.

### Key to Chinese species of *Stenomesius* Westood based on females

**Table:** 

1	Scutellum with sublateral grooves not meeting each other posteriorly	***S.guanshanensis* , sp. nov.**
–	Scutellum with sublateral grooves meeting each other posteriorly	**2**
2	Clava 3-segmented; axillae almost meeting medially	***S.japonicus* (Ashmead)**
–	Clava 2-segmented; axillae separated from each other	**3**
3	Midlobe of mesoscutum with 2 pairs of setae; axillae smooth	***S.maculatus* Liao**
–	Midlobe of mesoscutum with 3 pairs of setae; axillae finely reticulate	**4**
4	Mesoscutum with anterior half black and posterior half yellow, forewing with a line of setae in full length of costal cell on upper surface	***S.hani*, sp. nov.**
–	Mesoscutum dark brown, forewing with several setae on upper surface of costal cell distally	***S.harbinensis*, sp. nov.**

### Stenomesius guanshanensis sp. nov.

Taxon classificationAnimaliaHymenopteraEulophidae

817548A1-302E-5C69-AD24-04BA2C1B187F

http://zoobank.org/1BB67FF9-E5CE-4025-80C7-6D25C63BCC1D

Figures 1–6

#### Type material.

***Holotype***, ♀ [NEFU; on card], China, Jiangxi Province, Yichun City, Guanshan National Nature Reserve, 2.VIII.2018, Xiang-Xiang Jin and Wang-Ming Li, by sweeping.

***Paratype***: 1♀ [on slide], same data as holotype.

#### Diagnosis.

Lower face yellow; interantennal area yellowish; upper face and vertex black with area around eye margins yellow; antenna inserted at high above the lower eye margins and on the middle of between anterior ocellus and lower margin of clypeus. Pronotum with dorsal side black and lateral sides yellowish; mesoscutum, axillae and scutellum yellow or yellowish except about posterior 1/6 of scutellum black; midlobe of mesoscutum with 4 pairs of long setae; scutellum light alutaceous; dorsellum and lateral panels black; propodeum yellowish with carina brown.

#### Description.

**Female.** Length 2.0–2.2 mm. Lower face yellow; interantennal area yellowish; upper face and vertex black with area around eye margins yellow; scape yellow except apical half of dorsal surface pale brown, pedicel and flagellum dark brown; mandibles yellow with teeth brown; gena yellow; occiput black. Pronotum with dorsal side black and lateral sides yellowish; mesoscutum, axillae, and scutellum yellow or yellowish, except about posterior 1/6 of scutellum black; dorsellum and lateral panels black; propodeum yellowish, with carina brown; wings hyaline, with veins pale brownish yellow; legs pale yellow, with claws brown; gaster mainly yellowish with apex brown, anterior 1/3 of lateral margins, two spots on median part, and spots on lateral sides of T4–7 dark brown; ovipositor black.

***Head*** (Fig. 3) 1.5 × as wide as high in frontal view and 2.0 × as wide as long in dorsal view; frons and vertex smooth, shiny, with very sparsely scattered setae; POL 1.3 × OOL; eye with sparse short pubescence; malar sulcus present, shallow; malar space 0.24 × eye height; mandibles with 6 teeth; occiputal margin with dense setae. Antenna (Fig. 4) inserted at high above the lower eye margins and on the middle of between anterior ocellus and lower margin of clypeus; scape exceeding level of vertex and apical 2/3 with setae dorsally. Relative measurements (length : width): scape = 56 : 9; pedicel = 18 : 9; F1 = 28 : 8; F2 = 27 : 8; F3 = 29 : 8; F4 = 26 : 8; clava = 36 : 8 (excluding terminal spine).

**Figures 1–6.**
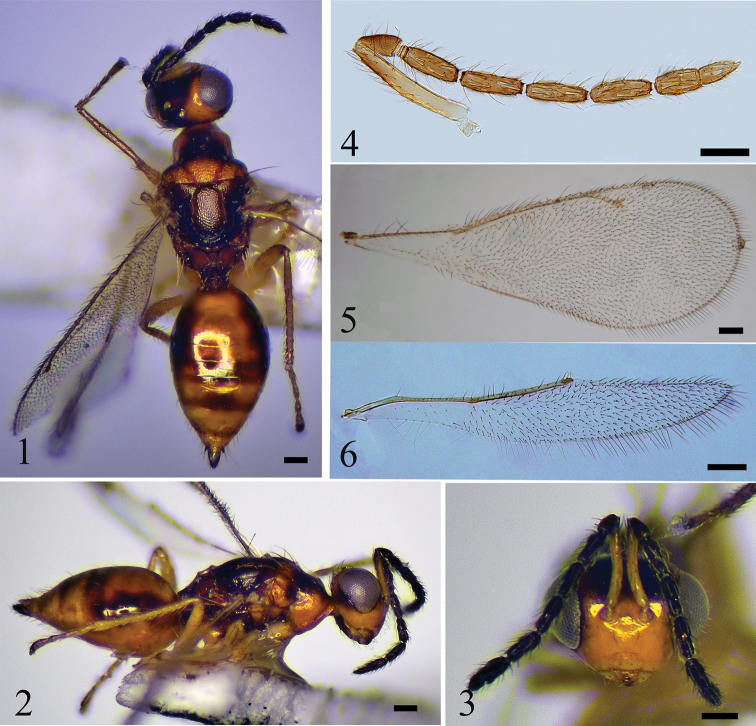
*S.guanshanensis*, sp. nov. **1–3** female, holotype **1** habitus in dorsal view **2** habitus in lateral view **3** head in frontal view **4–6** female, paratype **4** antenna **5** forewing **6** hind wing. Scale bars: 100 μm.

***Mesosoma*** (Fig. 1). Pronotum with a row of setae on anterior margin and 2 long pale yellow setae on posterior median margin; mesoscutum with anterior half reticulate and posterior half almost smooth; midlobe of mesoscutum with 3 pairs of long setae on median area and 1 pair of long setae on posterior area; axillae and scutellum shiny, light alutaceous; axillae well separated from each other; scutellum with 2 pairs of long setae; sublateral grooves not meeting each other medially on posterior margin; propodeum smooth, with plicae and H-shaped carina; each propodeal callus with 6 long setae; forewing (Fig. 5) 2.7 × as long as wide; speculum absent; hind wing (Fig. 6) about 6.2 × as long as wide; metatibial spurs shorter than corresponding basitarsomere. Relative measurements (length): SMV = 22; MV = 27; PMV = 11; STV = 8.

***Metasoma*** (Fig. 1). Petiole short, transverse; gaster ovate; 2.4 × as long as wide and 1.3 × as long as mesosoma; ovipositor exserted at apex of gaster.

**Male.** Unknown.

#### Host.

Unknown.

#### Distribution.

China (Jiangxi).

#### Etymology.

The specific name is derived from the name of the type locality.

### Stenomesius hani sp. nov.

Taxon classificationAnimaliaHymenopteraEulophidae

E343D290-0D1B-5070-B537-1B0AE10FC552

http://zoobank.org/B9CC3AE5-A995-47E1-B0E8-76B35BAAAEBD

Figures 7–12

#### Type material.

***Holotype***, ♀ [NEFU; on card], China, Heilongjiang Province, Mudanjiang City, Heixiazigou, 26–28.VIII.2015, Hui Geng, Yan Gao and Zhi-Guang Wu, by yellow pan trapping.

***Paratype***: 1♀ [on slide], China, Heilongjiang Province, Shangzhi City, Maoershan, 18.V.2018, Guang-Xin Wang, Ming-Rui Li and Ye Sai, by sweeping.

#### Diagnosis.

Lower face yellow; upper face dark brown except lower most about 1/6 yellow; vertex dark brown with 2 triangular, yellow spots; antenna inserted at middle of the face; scape yellow, with about apical half of dorsal surface brown; mesoscutum with anterior half black and posterior half yellow; midlobe of mesoscutum with 3 pairs of long setae; scutellar-axillar complex dark brown, metanotum black; propodeum yellowish brown.

#### Description.

**Female.** Length 1.7–1.9 mm. Lower face yellow; upper face dark brown except lower 1/6 yellow; vertex dark brown with a triangular, yellow spots at each side; scape yellow with about apical half of dorsal surface brown, pedicel and flagellum black; mandibles yellow, with teeth brown; gena yellow; occiput mostly dark brown. Pronotum yellow, with a dark brown spot in the middle; mesoscutum with anterior half black and posterior half yellow; axillae and scutellum dark brown; dorsellum and lateral panels black; propodeum brownish; Petiole dark brown; gaster mainly yellowish-brown, with anterior half of lateral margins dark brown, T3–5 each with a transverse dark brown stripe; ovipositor black. wings hyaline with veins pale brownish yellow; legs yellow, with claws brown.

***Head*** (Fig. 9) 1.4 × as wide as high in frontal view and 2.2 × as wide as long in dorsal view; frons smooth and shiny with a row of setae around eye margins; vertex smooth with scattered setae; POL 1.4 × OOL; eyes with sparse, short pubescence; malar sulcus present, shallow; malar space 0.38 × eye height; occiput with dense setae. Antenna (Fig. 10) inserted at middle of the face; scape exceeding level of vertex. Relative measurements (length : width): scape = 57: 8; pedicel = 16: 9; F1 = 26: 9; F2 = 27: 8; F3 = 27: 8; F4 = 27: 8; clava = 37: 10 (excluding terminal spine).

**Figures 7–12.**
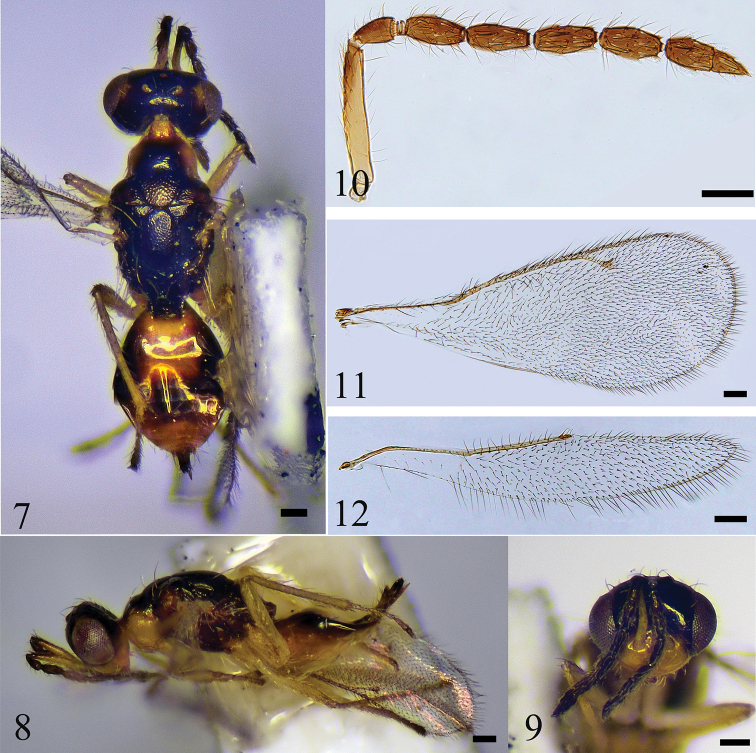
*S.hani*, sp. nov. **7–9** female, holotype **7** habitus in dorsal view **8** habitus in lateral view **9** head in frontal view **10–12** female, paratype **10** antenna **11** forewing **12** hind wing. Scale bars: 100 μm.

***Mesosoma*** (Fig. 7). Pronotum with a row of setae along anterior margin and 2 long, pale yellow setae medially along posterior margin; mesoscutum and axillae finely reticulate; midlobe of mesoscutum with 3 pairs of long setae; axillae well separated from each other; scutellum distinctly reticulate, with 2 pairs of long setae; sublateral grooves uniting posteriorly; propodeum smooth, with plicae and H-shaped carina; each propodeal callus with 7 long setae; forewing (Fig. 11) 2.7 × as long as wide; speculum absent; hind wing (Fig. 12) 7.2 × as long as wide; metatibial spurs about as long as corresponding basitarsomere. Relative measurements (length): SMV = 21; MV = 19; PMV = 7; STV = 5.

***Metasoma*** (Fig. 7). Petiole rugose, short, transverse; gaster ovate, 1.7 × as long as wide and 1.1 × as long as mesosoma; ovipositor exserted at apex of gaster.

**Male.** Unknown.

#### Host.

Unknown.

#### Distribution.

China (Heilongjiang).

#### Etymology.

The species is named for Prof. Hui-Lin Han of the Northeast Forestry University, Harbin, China.

#### Remarks.

*Stenomesiushani* is similar to *S.anati* Khan & Singh in sharing: antenna inserted in middle of face; face with dark brown infuscation above antennal torulus; anterior half of mesoscutum, scutellum, and axillae with dark brown infuscation. *Stenomesiushani* can be separated from the latter by the following combination of characters: scutellum with 2 pairs of setae (3 pairs in *S.anati*); distal 1/3 costal cell with setae (whole costal cell with setae in *S.anati*); gaster 1.1× as long as mesosoma (shorter than mesosoma in *S.anati*).

### Stenomesius harbinensis

Taxon classificationAnimaliaHymenopteraEulophidae

E6A3CCBA-9B92-5E49-B421-58104BB76AD5

http://zoobank.org/D045B6DE-2B79-44AE-B26E-51B2F153EC76

Figures 13–18

#### Type material.

***Holotype***, ♀ [NEFU; on card], China, Heilongjiang Province, Harbin City, Northeast Forestry University, 20–21.IX.2018, Ming-Rui Li, Wen-Jian Li, Jun-Jie Fan, Yu-Ting Jiang, Guang-Xin Wang and Jun Wu, by yellow pan trapping.

***Paratypes***: 1♀ [on slides], same data as holotype; 1♀ [on card], China, Heilongjiang Province, Shangzhi City, Maoershan, 18–19.V.2018, Ming-Rui Li, Guang-Xin Wang and Ye Sai, by yellow pan trapping.

#### Diagnosis.

Vertex black; occiput mostly black with two small, yellow spots near eyes margins; lower face yellow; upper face black; antenna inserted in above the lower eye margins; scape mostly yellow with about apical half of dorsal surface pale brown; pronotum with dorsal surface black and lateral sides yellowish; mesoscutum dark brown; midlobe of mesoscutum with 3 pairs of long setae; scutellar-axillar complex, metanotum, and propodeum black.

#### Description.

**Female.** Length 1.8–1.5 mm. Head black with area below antennal toruli and two small spots on vertex yellow; scape mostly yellow with about apical half of dorsal surface pale brown, pedicel, and flagellum dark brown; mandibles yellow, with teeth brown; gena yellow; occiput black. Pronotum with dorsal side black and lateral sides yellowish; mesoscutum dark brown; scutellar-axillar complex, metanotum, and propodeum black; wings hyaline, with veins pale brownish yellow; legs yellow with claws brown; gaster mainly black, with apex brown, anterior half with a median, large, yellowish-brown spot; ovipositor black.

***Head*** (Fig. 15) 1.3 × as wide as high in frontal view and about 2.0 × as wide as long in dorsal view; face smooth, shiny, with fine setae around eye margins; vertex with scattered setae; POL 1.3 × OOL; eye with sparse short pubescence; malar sulcus present, shallow; malar space 0.36 × eye height; mandible curve inwards and well developed; occiput with dense setae. Antenna (Fig. 16) inserted at above lower eye margins. Relative measurements (length : width): scape = 54 : 9; pedicel = 14 : 9; F1 = 22 : 10; F2 = 23 : 10; F3 = 22 : 10; F4 = 22 : 12; clava = 32 : 11 (excluding terminal spine).

**Figures 13–18.**
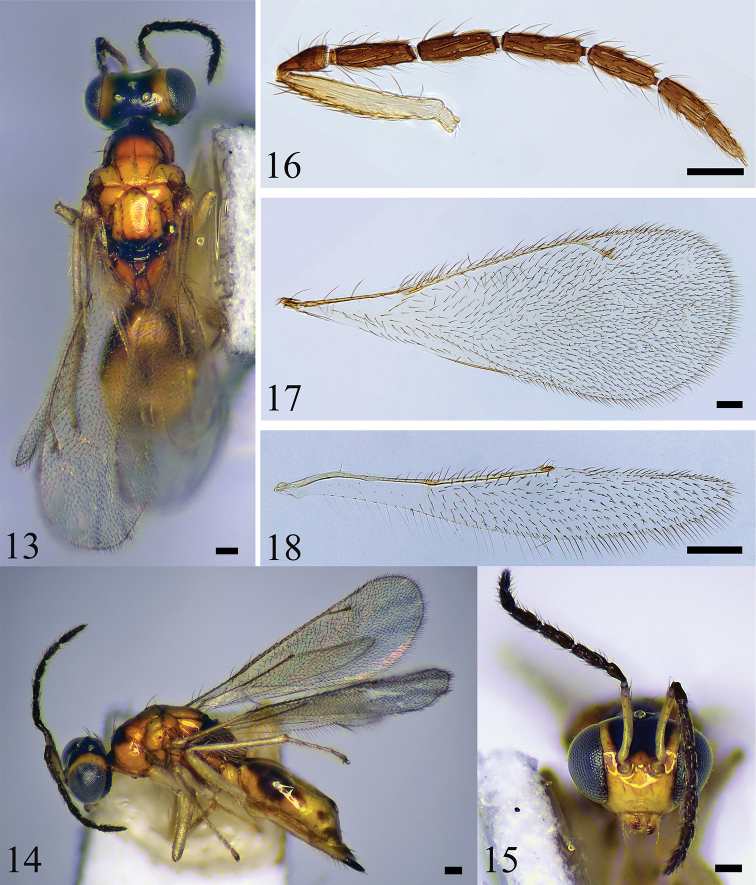
*S.harbinensis*, sp. nov. **13–15** female, holotype **13** habitus in dorsal view **14** habitus in lateral view **15** head in frontal view **16–18** female, paratype **16** antenna **17** forewing **18** hind wing. Scale bars: 100 μm.

***Mesosoma*** (Fig. 13). Pronotum with a row of setae along anterior margin and 2 long, pale yellow setae on posterior median margin; mesoscutum, axillae, and scutellum distinctly reticulate; midlobe of mesoscutum with 3 pairs of long setae; notauli curved in posterior part; axillae well separated from each other; scutellum with 2 pairs of long setae; sublateral grooves meeting each other medially on posterior margin; propodeum smooth, with plicae and H-shaped carina; each propodeal callus with 10 long setae; forewing (Fig. 17) 2.6 × as long as wide; speculum absent; hind wing (Fig. 18) about 5.8 × as long as wide; metatibial spur shorter than corresponding basitarsomere. Relative measurements (length): SMV = 36; MV = 41; PMV = 16; STV = 9.

***Metasoma*** (Fig. 13). Petiole short, transverse, about 1.2 × as long as wide in dorsal view; gaster ovate, 1.9 × as long as wide, and 1.1 × as long as mesosoma; ovipositor exserted at apex of gaster.

**Male.** Unknown.

#### Host.

Unknown.

#### Distribution.

China (Heilongjiang).

#### Etymology.

The specific name is derived from the name of the type locality.

## Supplementary Material

XML Treatment for Stenomesius

XML Treatment for Stenomesius guanshanensis

XML Treatment for Stenomesius hani

XML Treatment for Stenomesius harbinensis
